# A statistical shape model of soleus muscle morphology in spastic cerebral palsy

**DOI:** 10.1038/s41598-022-11611-z

**Published:** 2022-05-11

**Authors:** Salim G. Bin Ghouth, Sian A. Williams, Siobhan L. Reid, Thor F. Besier, Geoffrey G. Handsfield

**Affiliations:** 1 Auckland Bioengineering Institute, University of Auckland, Auckland, 1010 New Zealand; 2 Curtin School of Allied Health, Curtin University, Perth, WA 6845 Australia; 3 Liggins Institute, University of Auckland, Auckland, 1010 New Zealand; 4 School of Human Sciences (Exercise Science), University of Western Australia, Perth, WA 6009 Australia; 5 Department of Engineering Science, University of Auckland, Auckland, 1010 New Zealand

**Keywords:** Paediatric research, Computational models, Image processing, Skeletal muscle, Biomedical engineering

## Abstract

This study investigated morphological characteristics of the soleus muscle in cerebral palsy (CP) and typically developing (TD) cohorts using a statistical shape model and differentiated dominant features between the two cohorts. We generated shape models of CP and TD cohorts to characterize dominant features within each. We then generated a combined shape model of both CP and TD to assess deviations of the cohorts’ soleuses from a common mean shape, and statistically analysed differences between the cohorts. The shape models revealed similar principal components (PCs) with different variance between groups. The CP shape model yielded a distinct feature (superior–inferior shift of the broad central region) accounting for 8.1% of the model’s cumulative variance. The combined shape model presented two PCs where differences arose between CP and TD cohorts: size and aspect ratio of length–width–thickness. The distinct appearance characteristic in the CP model—described above—may implicate impaired muscle function in children with CP. Overall, children with CP had smaller muscles that also tended to be long, thin, and narrow. Shape modelling captures dominant morphological features of structures, which was used here to quantitatively describe CP muscles and further probe our understanding of the disease’s impact on the muscular system.

## Introduction

Cerebral palsy (CP) is a neurodevelopmental disorder arising from a non-progressive brain lesion that occurs in utero or soon after birth^1–3^. The brain lesion leads to progressive secondary musculoskeletal conditions affecting both movement and posture of children with CP^4,5^. The musculoskeletal conditions of CP start at an early age and progress through the lifespan, influencing quality of life and wellbeing^5,6^. These conditions arise from, among other things, impaired muscle in CP and may be influenced by altered development of skeletal muscles^3^. CP is the most common cause of physical disability in childhood with a prevalence of 1.5–2.5 per 1000 live births^5,7^. Although CP is a lifelong condition, the focus of clinical intervention and management are more commonly targeted during childhood, which is a period of rapid growth and development^8^. Understanding shape differences in the muscles during this period, a key time point for the manifestation of common musculoskeletal impairments in children with CP, is important to understand the way CP impacts the muscles compared to typical children of the same age. Understanding shape differences would further build upon prior work which has focused on differences in volumes, lengths, and cross-sections between populations.

Structural and architectural properties of the lower-limb skeletal muscles have been investigated in the past in children with CP. Common measurements include volume, muscle length, fascicle length, anatomical cross-sectional area, physiological cross-sectional area (PCSA), and pennation angle^9–14^ (see, e.g.^13^ for a systematic review). One particular muscle of interest in the lower-limb is the soleus, which is essential for both stance and power generation in walking, is often implicated in impaired gait patterns, and has a commonly altered muscle architecture in people with spastic CP^11,15^. Previous works have reported impairments in soleus muscle volume^9,11,12^, cross-sectional areas^10–12^, and muscle length^10–12^ among children with spastic CP. Deficits of size and length are clearly a common effect of spastic CP on the muscles; however, it is unknown how shape and morphology differ between CP and typically developing (TD) individuals. Understanding shape differences may offer more insight into the ways CP affects the muscles, and whether there are patterns of abnormal muscle shape which are common in CP. To the authors’ knowledge, quantitative descriptions of shape differences have not been reported for CP muscles, leaving this a compelling area for exploration.

Statistical shape modelling (hereafter, also referred to as shape modelling) is a computational method to reduce high dimensions of complex, anatomical geometries into a smaller set of parameters, or principal components (PCs) which represent the dominant features of the anatomy across a population or subpopulation^16,17^. Shape modelling has been used to investigate the variation of anatomical structures in orthopedics^18–24^, cardiology^25,26^, and neurology^27,28^. It has been used for purposes like morphometric analysis^18–21^, extended to study the relationship between anatomical features and parameters of interest (e.g. pathology or sex)^22–24,28^, and used to inform the design of a classifier (e.g. Fisher’s Linear Discriminant)^27^. Shape models have not, as yet, been used to investigate the morphological variation of lower limb skeletal muscles, and such an approach could prove insightful for understanding altered musculature in CP.

The objectives of this study were to generate a statistical shape model of the soleus muscle to (1) investigate the morphological variability within a cohort of children with CP and a TD cohort; and (2) identify the differences between children from CP and TD cohorts. We hypothesized that the CP and TD cohorts will be distinguishable via one or more PCs of the shape models representing the dominant morphological and appearance characteristics of each population.

## Methodology

**Figure 1:**
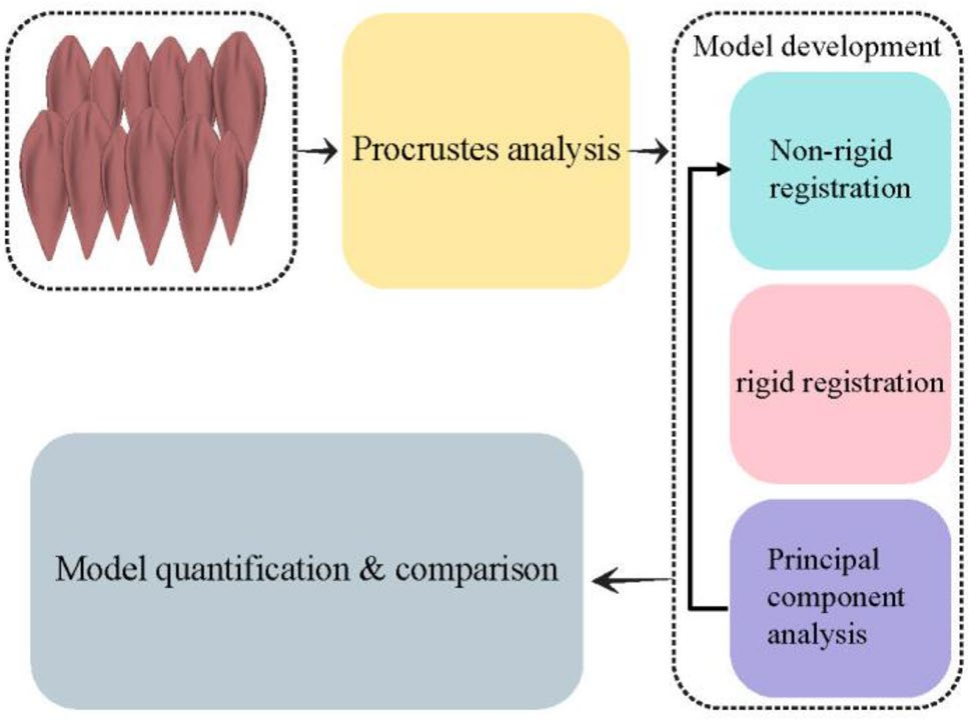
The flowchart of the model generation and quantification process.

Our methods comprised segmentation of images from CP and TD cohorts, statistical shape model development, and model quantification and comparisons (Fig. 1) which we describe in detail below.

### Participant characteristics and imaging protocols

Statistical shape models were developed using MR images of the soleus muscle of children and adolescents with CP (n_CP_ = 57) and TD children and adolescents (n_TD_ = 17) obtained from five previous studies: Handsfield et al*.*^11^, Sahrmann et al*.*^12^, Williams et al*.*^29^, Reid et al*.*^30^, and Alexander et al*.*^31^. All five prior studies were approved by and complied with respective institutional ethics for human subjects’ research: the institutional review board of the University of Virginia, the University of Auckland Human Participants Ethics Committee, and the Princess Margaret Hospital/Perth Children’s Hospital Research Ethics Committee. All data acquisitions were performed in accordance with the relevant guidelines and regulations, as were the transfer and use of these de-identified data by our group. All participants and/or parents and guardians provided informed consent. The MR images from previous studies were manually segmented slice by slice by research investigators trained in segmentation. All segmentations were processed and inspected for consistency across the studies; surface meshes were exported as stereo-lithography (STL) files from MeshLab software (Version 1.3.4, http://meshlab.sourceforge.net)^32^. For this study, we used muscles from the paretic limbs of participants with hemiplegic CP (n = 15) and from the most affected limbs of participants with diplegic CP (n = 42). Left-leg segmentations (26 out of 57 children and adolescents with CP, 3 out of 17 TD children and adolescents) were reflected against the sagittal plane so all shape models here are of the right limb muscles and reflected left limb muscles.

The characteristics of CP and TD participants (mean ± SD [range] of sex, age, mass, height, and BMI) are provided in Table 1. Participants’ characteristics were statistically evaluated using t-tests to determine differences between the CP and TD cohorts. Where data were not normally distributed, we used a Kruskal–Wallis test. Participants had no intervention in the six months prior to the scanning sessions. The participants with CP were ambulatory ranging from level I to III of the Gross Motor Function Classification System (GMFCS) (GMFCS I = 31, GMFCS II = 19, GMFCS III = 7). Of the 57 participants with CP, 54 participants presented spasticity; spasticity was not reported for three participants, but presentation and musculature were consistent with spasticity. Seven participants with CP had a prior history of lower-limb orthopedic surgeries targeting muscles; surgical history data were not available for nine participants with CP. A sub-cohort of the CP participants, referred to here as CP_sub_, matching the imaging protocols and participant characteristics of the TD cohort was selected for the combined model to investigate the deviation of the soleus muscles of each cohort from a common mean shape. The imaging protocols of the collected MRI data used in this study are presented in Table 2.

**Table 1:** Participant characteristics.

The cohort	Gender (M/F)	Age^a^ (years)	BM1^a^ (kg/m^2^)	Height^a^ (m)	Mass^a^ (kg)
CP_total_ (n = 57)	38/20	9.5 ± 3.2^5–17^	18.0 ± 4.1 [12.8–31.5]	1.37 ± 0.81 [1.04–1.75]	35.1 ± 15.9 [15.4–96.1]
CP_sub_ (n = 19)	13/6	12.3 ± 2.6^8–17^	20.0 ± 5.1 [13.1–31.5]	1.50 ± 0.17 [1.14–1.75]	47.1 ± 19.6 [17.0–96.1]
TD (n = 17)	13/4	12.1 ± 2.8^7–17^	20.1 ± 4.2 [14.9–29.7]	1.55 ± 0.17 [1.23–1.78]	50.2 ± 18.6 [22.6–83.5]
*p*-value (CP_total_ vs TD)	–	0.003	0.034	0.0004	0.002
*p*-value (CP_sub_ vs TD)	–	0.87	0.96	0.38	0.63

^a^The table shows the mean ± SD [range] of the participant characteristics. CP_total_ here refers to the entire CP cohort in this study. CP_sub_ is a subset of 19 participants where all of the CP_sub_ was imaged with similar MRI protocols, which are similar to the TD cohorts’ MRI protocols.

**Table 2:** Imaging protocols and specifications.

Study reference	Scanner	Sequence	Parameters		
			TE/TR (ms/ms)	FOV (mm × mm)	Spatial resolution (mm × mm × mm)
Handsfield et al.^16^	3T Siemens Trio	2-D multi-slice gradient-echo pulse with an interleaved spiral k-space trajectory	3.8/800	400 × 400	1_._1 × 1.1 × 5^a^
Sahrmann et al.^17^	3T Siemens Skyra	High-resolution 3D T1 VIBE Dixon	5.22/10.4	128 × 228 × 384	0.8 × 0.8 × 0.8
Williams et al.^35^	1.5T Siemens Sonata Maestro	Axial spin-echo TI-weighted	13/572	280 × 280^b^	1.1 × 1.1 × 5^a^
Reid et al.^36^	1.5T Siemens Sonata Maestro	Axial spin-echo T1-weighted	13/572	280 × 280^b^	1.1 × 1.1 × 5^a^
Alexander et al.^37^	1.5T Siemens Sonata Maestro	Spin-echo TI-weighted	13/572	333.1 × 333.1^b^	1.3 × 1.3 × 5^a^

^a^Slice thickness for 2D scanners.

^b^FOV varied by anatomical location. We report here the maximum in each direction and the interpolated spatial resolution.

### Development of statistical shape models

Variations of high-dimensional muscle morphology and appearance in CP and TD cohorts were investigated using a statistical shape modelling framework developed by Zhang et al*.*^20^. Each surface mesh of the data sets was defined by a shape vector *S* in the shape space including a specific number of surface vertices *v* mapped at corresponding *x*, *y*, and *z* coordinates across the meshes.

1 $$S_{i} = \left[ {\left( {v_{jx} ,v_{jy} ,v_{jz} } \right)} \right].$$

Each shape is represented by a number of points (i.e., vertices) describing the shape surface in 3-dimensional space, and each point is described by three coordinates (x, y, and z). Equation (1) shows a representation, i.e., geometric characteristics, of each shape vector where i = 1…m (m is the number of shapes in the data set) and j = 1…n (n is the number of vertices in each surface mesh). An average TD surface mesh was selected as a registration reference for both cohorts. The surface meshes of both groups were fitted against the reference mesh (non-rigid registration) using radial basis functions^20^. The fitted meshes were aligned (rigid registration) based on their center of mass and principal axis of inertia coordinate system to remove translation and rotation misalignment. A dimensionality reduction and eigen-analysis method—principal component analysis (PCA)—was applied to generate a mean shape, refer to (2), and a covariance matrix representing each mesh in the data set. The eigen-analysis of the PCA yielded a combination of a mean shape, $\stackrel{\mathrm{-}}{\text{S}}$, eigenvectors b and corresponding eigenvalues P that can approximate each shape in the data set, refer to (3). The generated mean shape was used as a reference in an iterative refitting process of the data set until the fitting accuracy (RMS) was brought down to 0.86 mm and 0.87 mm for CP and TD cohorts respectively^16^.

2 $$\overline{S} = \frac{1}{m} \mathop \sum \limits_{i = 1}^{m} S_{i} ,$$

3 $$S_{i} = \overline{S } + Pb.$$

Ultimately, morphological (non-normalized) and appearance (normalized) statistical shape models were generated independently for each cohort (children and adolescents with CP, CP_total,_ and TD children and adolescents, TD) to characterize the anatomical variations within each of the two cohorts, i.e. intra-cohort variability. A combined statistical shape model (CP_sub_ and TD) was generated to study the variations between the two cohorts, i.e. inter-cohort variability. The appearance model (representing the normalized model) was generated by normalizing the data set using a least-squares minimization method, which minimizes the least-squares distance between a reference muscle and the remaining data set, implemented in the aforementioned framework^20^. We selected the first 9 eigenmodes, here the PCs, as a sufficient number of PCs that can compactly describe a high percentage of the cumulative variance—96% and 98% of CP and TD models, respectively—of the morphological models. The anatomical variation, i.e., morphology and appearance, within each cohort was quantitatively characterized. The registration quality of the registered surface meshes was evaluated, and the overall model quality was evaluated by a leave-one-out cross validation using partial least-squares regression.

### Comparison of statistical shape models

Initially, the mean soleus muscle of the TD cohort was mapped onto the mean soleus muscle of the CP cohort for both the non-normalized and normalized models. Then, color-coded difference maps were quantitatively assessed by calculating the Hausdorff distance between the corresponding nodes. To determine similarity between CP and TD cohorts, a sub-cohort of the CP participants was used, CP_sub_ (n = 19), from the studies Handsfield et al*.*^11^ and Sahrmann et al*.*^12^. The sub-cohort matched the TD cohort in terms of imaging protocol and participant characteristics (gender, age, BMI, height, and mass; see Table 1). This minimized any potential bias introduced to the combined models for the comparison purpose. Combined models of CP and TD were then generated. Surface meshes of the data set were assigned a ‘soleus weight’ that described the surface mesh along each principal component of the shape model. To compare the complex shape features of the CP and TD cohorts, the soleus weights of the combined shape models—both non-normalized and normalized—were obtained for each surface mesh along each principal component. Weights of size characteristics were extracted from the non-normalized shape models, and weights of appearance characteristics were extracted from the normalized shape models. The extracted weights were confirmed to meet the t-test criteria: normality distribution, variance equality, and exclusion of outliers. Normality distribution was evaluated by Shapiro–Wilk test, and Q–Q plot; variance equality was evaluated by Levene’s test. A t-test with Holm–Bonferroni correction was performed for statistical analysis of the appearance characteristics via soleus weights of CP and TD morphological and appearance characteristics. For soleus weights violating the t-test criteria, the non-parametric Kruskal–Wallis test was performed.

## Results

**Figure 2:**
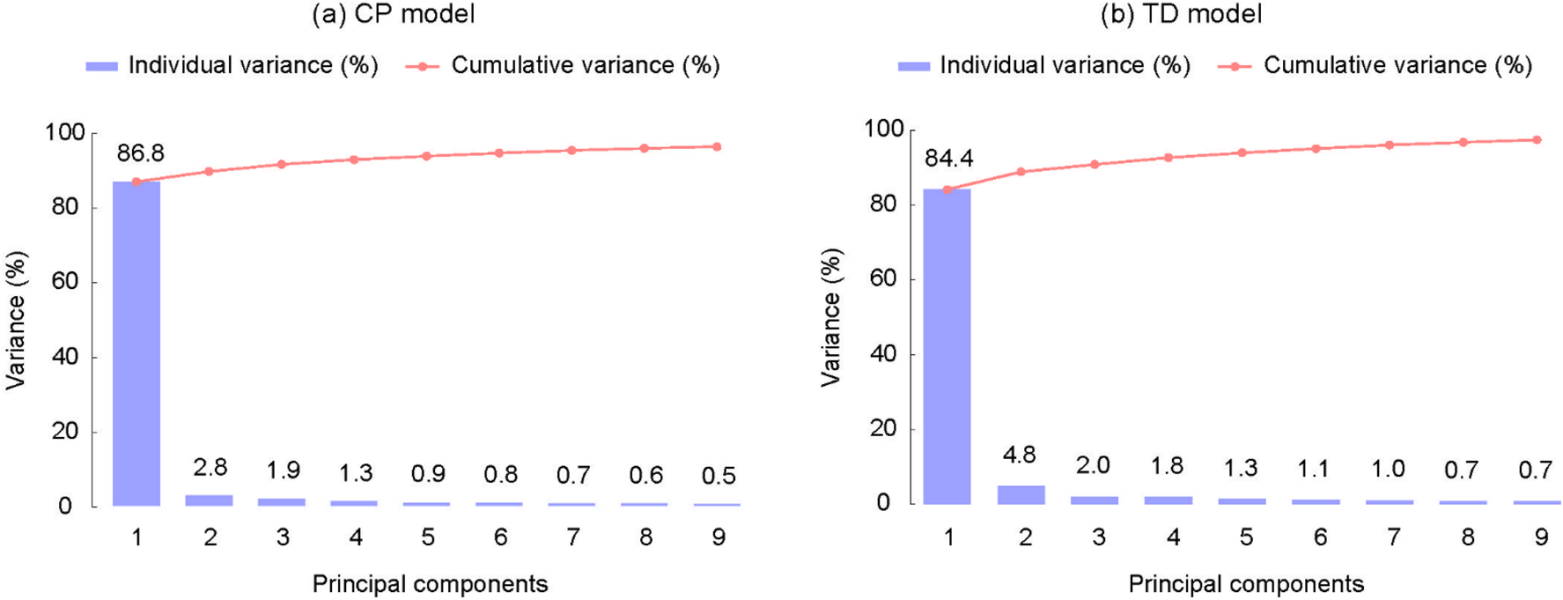
The first 9 PCs of the CP and typically developed (TD) morphological models and their cumulative variance.

The first nine principal components (PCs) of the CP cohort accounted for a cumulative variance of 96%, while the first five PCs accounted for 94% (Fig. 2a). Likewise, the cumulative variance of the first 9 PCs of the TD cohort accounted for 98%, while the first 5 PCs accounted for 94% (Fig. 2b), implying that 5 PCs can be used to describe over 90% of variance for both cohorts. The model quality tests using a leave-one-out technique, i.e., partial least squares regression, yielded RMS error of 0.85 mm and 0.85 mm for the CP and TD models, respectively, indicating a high degree of fidelity of the shape models.

### Intra-cohort variability characterization

#### Variability characterization within CP cohort

##### Morphological model of CP soleus muscle

**Figure 3:**
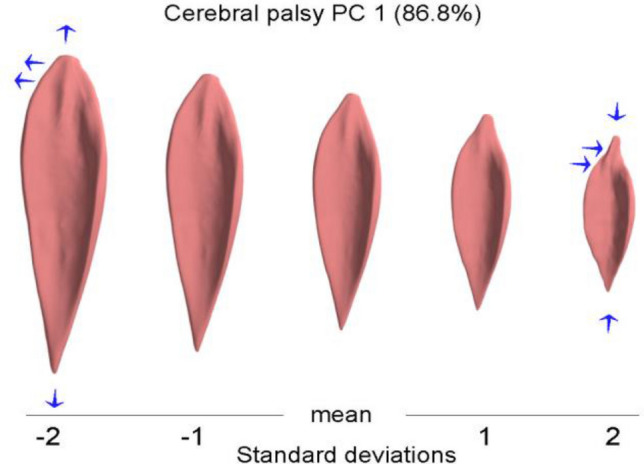
The variation of the CP soleus muscle along the first principal component (size). The blue arrows indicate the change of the muscle shape. The principal component can be described physically as the combination of two features: variation in overall size and a change of the muscle width at its proximal extremity.

The first PC of the morphological, i.e. non-normalized, model of the CP cohort accounted for 86.8% of the total variance. This PC can be described physically as the combination of two features: variation in overall size and a change of the muscle width at its proximal extremity (Fig. 3). Thus, these features together describe the vast majority of how soleus shape varies in the CP cohort. Widening of this proximal extremity is present at one end of the range of muscle variation and narrowing of this extremity is present at the other end of the range (see Fig. 3).

##### Appearance model of CP soleus muscle

**Figure 4:**
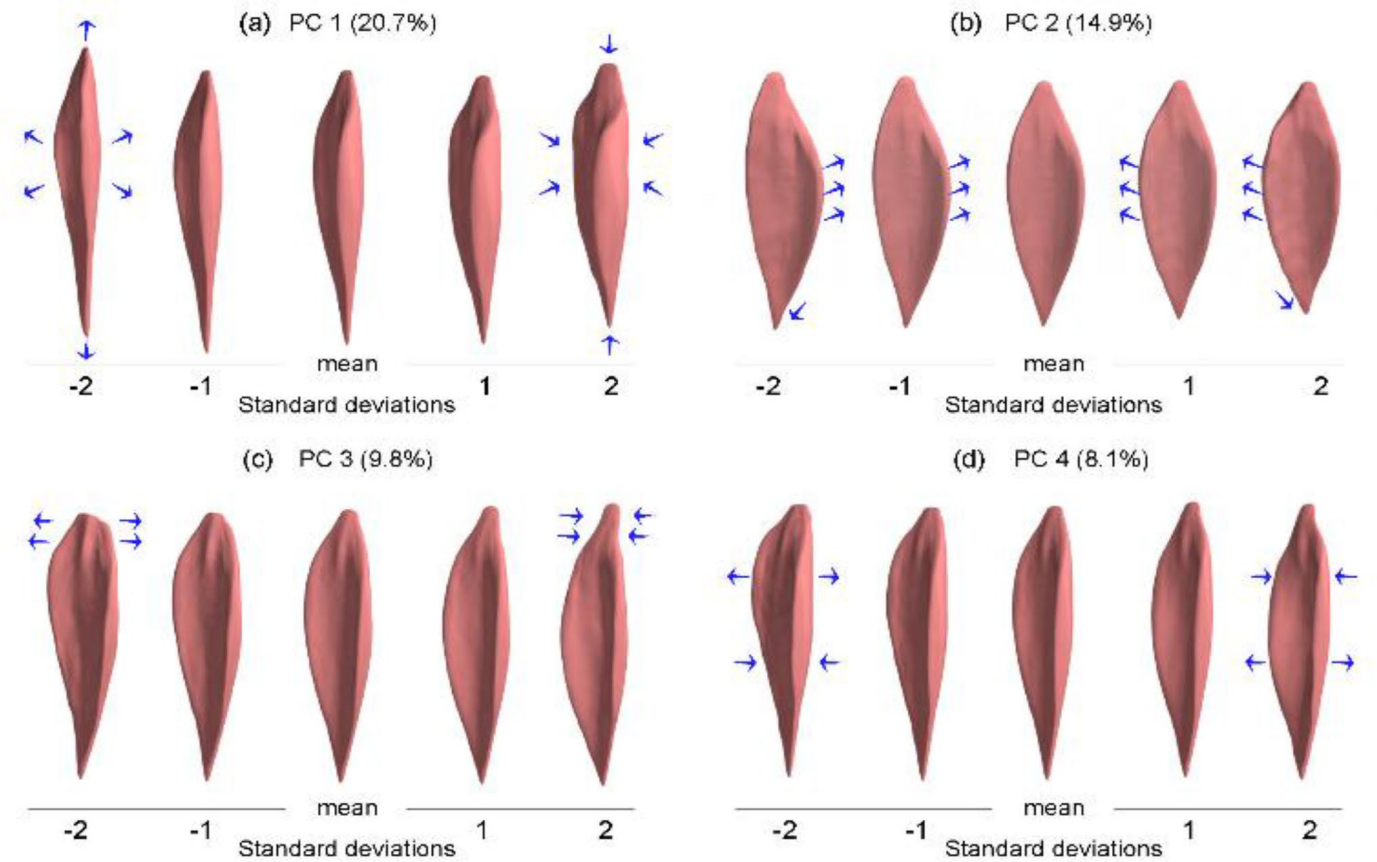
The variations of the soleus muscle along the principal components of the CP appearance (normalized) model and their variance percentages. The blue arrows indicate the change of the muscle shape. The first principal component (**a**) can be described as a change of the muscle length combined with an inverse change of the muscle thickness and width. The second principal component (**b**) represents a widening of the curved marginal aspect of the soleus. The third principal component (**c**) can be described as a change in the width of the proximal extremity of the muscle. The fourth principal component (**d**) represents a superior-inferior shift of the broad section of the muscle toward the muscle distal extremity.

The first PC of the appearance, i.e., normalized, model accounted for 20.7% of the total variance in CP muscles. Physically, this PC represents a change of the muscle length combined with an inverse change of the muscle thickness and width, e.g., ‘long-and-thin’ vs. ‘short-and-thick’ (Fig. 4a). This may be related to overall strength capacity and muscle length differences between participants.

The second PC accounted for 14.9% of the total variance. This PC represents a widening of the curved marginal aspect of the soleus, where there is a broader curve on the medial side for one end of the range, and a broader curve on the lateral side for the other end of the range (Fig. 4b). In other words, this PC relates to which side of the muscle displays the broad, curved marginal aspect.

The third PC accounted for 9.8% of the variance and can be physically described as a change in the width of the proximal extremity of the muscle (Fig. 4c).

The fourth PC accounted for 8.1% of the variation. Physically, this PC represents a superior-inferior shift of the broad section of the muscle toward the muscle distal extremity (Fig. 4d). We hypothesize that this may be related to adaptations after surgery, more on this in the “Discussion” section.

#### Variability characterization within TD cohort

##### Morphological model of TD soleus muscle

**Figure 5:**
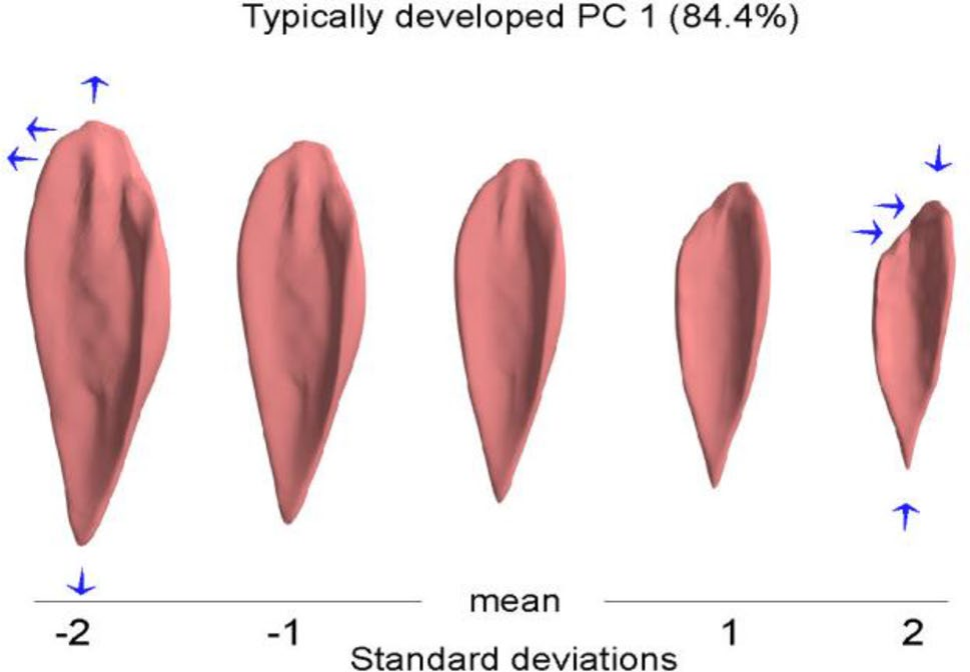
The variation of the TD soleus muscle along the first principal component (size). The blue arrows indicate the change of the muscle shape. The principal component can be described physically as the combination of two features: variation in overall size and a change of the muscle width at its proximal extremity.

The first PC of the morphological, i.e., non-normalized, model of the TD cohort accounted for 84.4% of the total variance. This PC can be described physically as the combination of two features: variation in overall size and a change of the muscle width at its proximal-medial extremity (Fig. 5). This is consistent with the first PC in the CP cohort, indicating similarity in the modes of variations between both cohorts. Widening of this proximal extremity is present at one end of the range of muscle variation and narrowing of this extremity is present at the other end of the range (see Fig. 5).

##### Appearance model of TD soleus muscle

**Figure 6:**
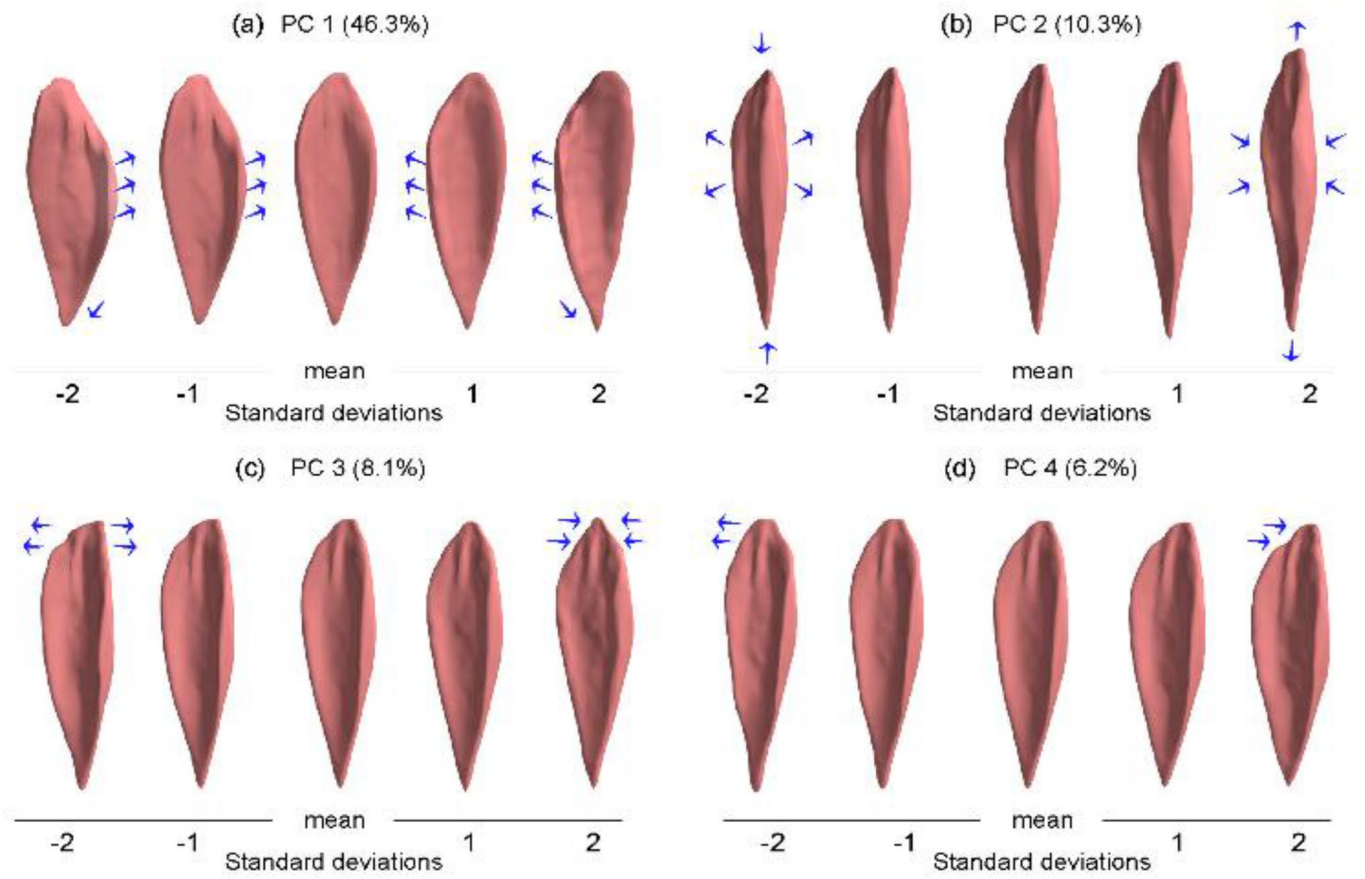
The variations of the soleus muscle along the principal components of the TD appearance (normalized) model and their variance percentages. The blue arrows indicate the change of the muscle shape. The first principal component (**a**) can be described by a widening of the curved marginal aspect of the soleus. The second principal component (**b**) represents a change of the muscle length combined with an inverse change of the muscle thickness and width. The third principal component (**c**) can be described as a change of the width of the proximal extremity of the muscle. The fourth principal component (**d**) represents a change at the proximal attachment region of the muscle.

The first PC of the TD appearance, i.e., normalized, model accounted for 46.3% of the total variance. This PC represents the previously described widening of the curved marginal aspect of the soleus, where there is a broader curve on the medial side for one end of the range, and a broader curve on the lateral side for the other end of the range (Fig. 6a). It is unclear the functional implication of this PC, but it mirrors the second PC of the CP cohort.

The second PC accounted for 10.3% of the total variance. As was the case in the CP model, this PC represents a change of the muscle length combined with an inverse change of the muscle thickness and width, e.g., ‘long-and-thin’ vs. ‘short-and-thick’ (Fig. 6b). This may be related to overall strength capacity and muscle length differences between participants and mirrors the first PC in the CP cohort.

The third PC accounted for 8.1% of the variance and can be described as a change of the width of the proximal extremity of the muscle (Fig. 6c).

The fourth PC accounted for 6.2% of the variation. Physically, this PC represents a change at the proximal attachment region of the muscle (widening at one end of the range of muscle variation and narrowing at the other end of the range of muscle variation) (Fig. 6d).

### Inter-cohort variability characterization

**Figure 7:**
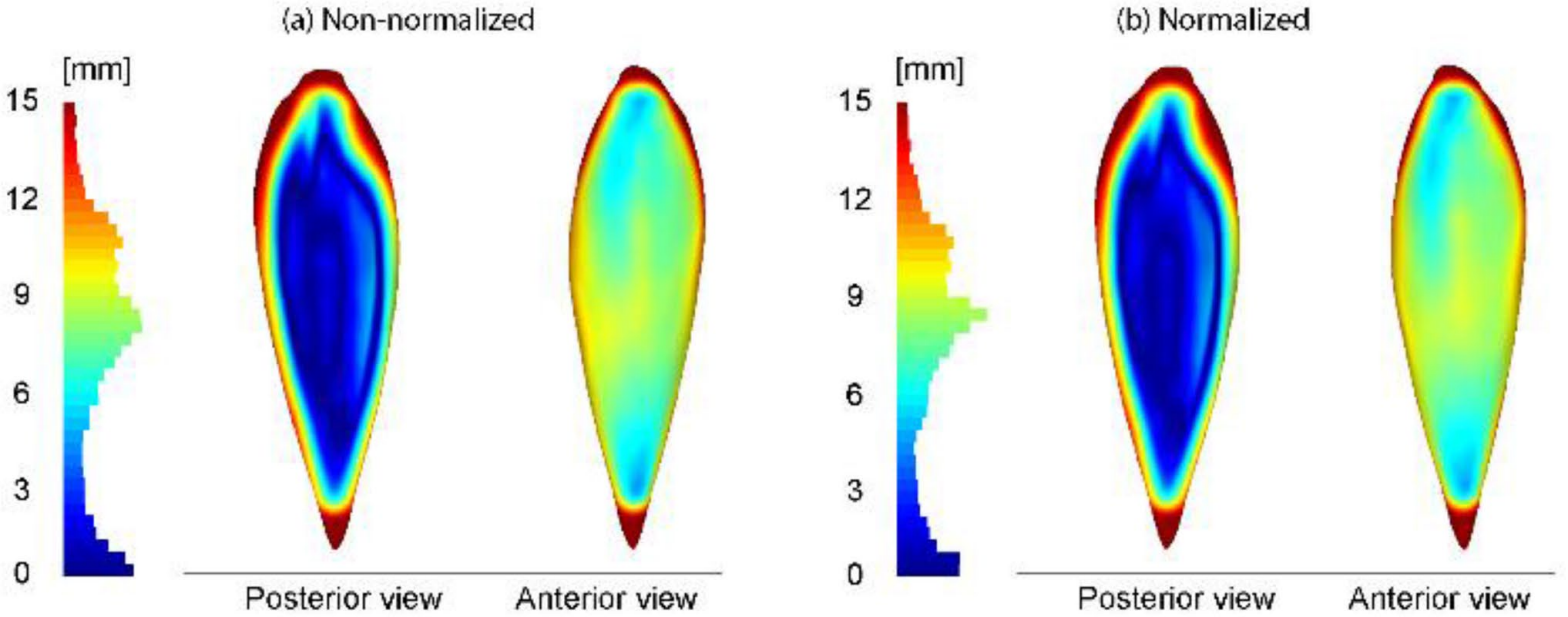
The non-normalized and normalized mean soleus muscles of the TD cohort mapped on the non-normalized and normalized CP-cohort mean soleus muscles. Blue regions indicate a minimal size difference between TD and CP (a TD muscle being similar in size to a CP muscle).; whereas red regions indicate maximal size difference between TD and CP (a TD muscle being greater in size than a CP muscle).

The primary difference between the mean soleus morphology of TD and CP cohorts was that the mean soleus muscle of the TD cohort is expanded in the proximal–distal and medial–lateral direction (Fig. 7). The differences were smaller in the anterior–posterior direction of the muscle. However, regardless of the Procrustes analysis, i.e., size normalization, the difference between the mean soleus muscle of the CP and TD cohorts was the same.

When investigating the deviations of the CP and TD soleus muscles from a common mean soleus muscle, the PCs corresponding to size and the aspect of length–width–thickness were different (*p*-value = 0.003, 0.021 respectively).

## Discussion

In this study, we used statistical shape modelling to assess the dominant shape characteristics of adolescent soleus muscles with and without cerebral palsy. We found that nine PCs were sufficient to describe the shapes for both CP and TD models, accounting for 96% and 98% of the variance, respectively. A reduced number of PCs, in this case five, was sufficient to account for 94% of the variance for both cohorts, indicating that the models are compact. The reported RMS values of the leave-one-out method were below the MRI resolution of the data set (see Table 2) which confirmed excellent generality and specificity measures in our shape models.

The intra-cohort variability indicated that size variations dominated the first principal component for both CP and TD models. This was expected and consistent with various statistical shape models of bones reported in the literature^18–20,22,23^. The reported variance (84.4% and 86.8%) of size variations in our study was greater than what is found in shape models of bones of an adult population (typically between 50 and ~ 80%)^19–21,23^. The age range of our data was between 5 and 17 years old, which is a period of considerable and variable growth in children and adolescents. In addition, it is important to note that size variation in the CP cohort is slightly greater than the TD cohort. This may represent the size variation of the GMFCS levels within the CP cohort.

When size differences were eliminated via normalization, the appearance variations of the CP and TD models were described by a similar set of PCs; in fact, only one PC out of four differed between the cohorts. The PCs which were common between the cohorts were (1) the aspect of length–width–thickness (long-thin vs short-broad), (2) the side of the muscle in which the marginal compartment was broad (lateral vs medial), and (3) the width of the proximal attachment region of the muscle (narrow vs wide).

The variance of each of these appearance characteristics differed between CP and TD cohorts, indicating that they explain different percentages of the variation within each cohort. The length–width–thickness PC accounted for 20.7% in the CP model, whereas it accounted for 10.3% in the TD model. It should be noted that reduced muscle thickness is an often reported observation in CP; our results are consistent with this, suggesting this varies in the CP population and may reflect degrees of severity of CP as it affects the soleus. Ohata et al*.*^33^ reported a difference in thickness of quadriceps femoris muscle thickness between degrees of CP severity (levels IV and V of the GMFCS). Chen et al*.*^34^ reported a difference in the soleus muscle thickness between cohorts of children and adolescents with diplegic CP and TD children and adolescents. They reported reduced thickness in the soleus muscle of children and adolescents with diplegic CP compared to TD, although they found no differences among the hemiplegic children and adolescents. The fourth principal component of the TD model (widening vs narrowing of proximal region) displayed less variance as a PC in the CP model (< 5%).

The remaining PC in the CP model is unique, i.e., it is not present as a dominant shape descriptor in the TD model. This PC represents a superior-inferior shift of the broad section of the muscle within the CP cohort. This PC accounted for 8.1% of the total variance in the CP cohort, which is considerable and bears questioning what might account for this appearance difference between the two cohorts. It has been reported in the literature that shortening of the muscle–tendon unit is associated with equinus contracture, a common gait impairment in children and adolescents with CP^35–37^. One treatment that has been used for equinus contracture in children and adolescents with CP is the Achilles tendon lengthening (ATL) surgery. Approximately 36% of the treated children and adolescents with ATL experience over-lengthening of the muscle–tendon unit which may lead to the development of crouch gait; 41% of ATL recipients experience recurrence of equinus^35,38^. In our dataset, 7% of the participants with CP had previously undergone an ATL intervention. We posit here that this surgery may account for a superior shift of the broad section of the soleus muscle, and that this effect of surgical history may influence this appearance characteristic in the CP model. ATL is reported to be associated with sarcomere lengthening and reduced production of force^39^. Future modelling work may illuminate whether the shape changes described by the aforementioned PC account for some of this sarcomere lengthening and force reduction. Further work on the phenomenon of postoperative muscle adaptation could improve surgical planning for children and adolescents with CP. We contend that shape modelling could be an advantageous component of this work.

The inter-cohort variability revealed that the mean soleus muscle of the TD cohort is an expanded version of the CP mean soleus muscle in the proximal–distal and medial–lateral direction, but not the posterior–anterior direction (see Fig. 7). This was true for both the non-normalized and normalized mean soleus muscles. In addition, the deviations of the soleus muscles of both cohorts from a common mean soleus muscle along each PC were different. Even though we see common variations in CP and TD cohorts, the magnitude of these variations are not identical. The assessment of the soleus muscles’ deviations from the common mean muscle yielded a difference between the two cohorts in the PCs of size and aspect of length–width–thickness. It has been reported that the soleus muscle of CP cohorts is different to TD in terms of volume, muscle thickness and anatomical cross-sectional area, all of which are supported by our results^9–12^. Soleus muscle length differences are not uniformly agreed upon^10–12^. Previous muscle architecture assessments in CP have used conventional definitions of the important parameters for understanding biomechanical differences in CP muscles, namely PCSA, fascicle length, and pennation angle. By investigating overall quantitative shape differences in the present work, it may be possible to elucidate abnormalities in muscle growth, gait patterns which affect regions of the muscle, or, as we discuss above, muscle-tissue adaptation resulting from surgical interventions. Additionally, it is possible that shape differences in the soleus muscle may associate with gross functional outcome such as gait or other movements. Neural and motor impairments in children and adolescents with CP may also contribute to impaired shape characteristics and are known to impact muscle strength^40^.

There are a few limitations to be considered in this study. The sample size of the data set included 57 participants with CP and 17 TD participants. This sample size enabled us to capture dominant features of both cohorts with excellent model quality measures. However, a greater sample size will provide more confidence that our results can be generalized to a larger population. The majority of participants with CP in this study presented spastic type CP, which may introduce a non-voluntary contraction of the soleus muscle or surrounding muscles while scanning. The participants were all scanned at rest, with their ankle and knee joints in a relaxed and neutral position to minimize muscle contractions. In addition, the soleus muscle is a deep muscle which reduces the possibility of any external pressure affecting the muscle morphology during scanning. Left leg soleus muscles were reflected against the sagittal plane to create morphological consistency in our models. This is based on our assumption that left leg muscles are, morphologically, mirrored right leg muscles.

In this study, we generated population-based statistical shape models of the soleus muscle in CP and TD children and adolescents. The CP cohort exhibited a PC that displayed a superior-inferior shift of the broad section of the muscle compared to the TD muscle. This feature accounted for 8.1% of the cumulative variance of the normalized CP model. Given that some of our participants with CP had received an ATL surgery prior to their participation in the study, which is associated occasionally with muscle over-lengthening and sarcomere lengthening^35,38,39^, we hypothesize a potential relationship between ATL surgery and the distinctive PC of the CP model; more work is needed to explore this link. In addition, a combined model was generated for both CP and TD cohorts to investigate the deviations of the soleus muscle of each cohort from a common mean soleus shape. We found that the PCs of size and aspect of length–width–thickness were different between CP and TD cohorts—smaller in children and adolescents with CP—which was consistent with literature. Biomechanical modelling of the morphology and appearance differences between CP and TD cohorts will help interpret the functional impairments and gait patterns in children and adolescents with CP. Shape modelling data are available upon request.
